# A Treatise on Fever

**Published:** 1833

**Authors:** 


Art. IV.—*A Treatise on Fevers.* By Southwood Smith, M. D. Physician to the London Fever Hospital. Philadelphia: Carey & Lea—pp. 450, octavo.

*Clinical Illustrations of Fever, comprising a Report of the cases treated at the London Fever Hospital, in* 1828 *and* 1829. By Alexander Tweedie, M. D., Physician to the London Fever Hospital, &c. &c. Philadelphia: Carey & Lea—pp. 150, octavo.

Fevers, from the earliest records of medicine until the present day, have formed the principal outlet of human life. They have, accordingly, received from physicians a degree of attention corresponding to their importance. From Hippocrates down to the numerous teachers of medicine who enlighten and adorn the age in which we live, almost every individual who has endeavored to establish a reputation by his writings, has offered to the world his disquisitions upon the causes and nature of fever, and the results of his experience in its treatment.

Of the numerous writers who have distinguished themselves in this department of professional science, few have enjoyed greater opportunities for observation, and few have the appearance of having devoted themselves to their task with a more patient spirit of investigation, than the gentlemen whose works are now under consideration.

The London Fever Hospital is capable of containing about 60 patients, and there pass through the wards annually from six to seven hundred patients. Two physicians are attached to the Institution, under whose care the patients are placed alternately, in the order in which they are admitted: there is one assistant physician whose duty it is to perform the duty of the ordinary physicians when either of them may be incapable of attending; and there is besides, a medical officer resident in the house. A history of each case, containing an account of the age, occupation, and residence of the patient, together with as full a statement of the symptoms of the disease, and of the order of their succession, as can be obtained, is entered in the journal by the resident medical officer. Each of the ordinary physicians attends daily, and enters in his journal a daily report of each of his own cases. The resident medical officer goes round the wards twice a day, to observe if any change requiring attention may have taken place in any patient, and all such events, with the modifications of treatment they have required, are entered in the journals. Every case that terminates fatally is examined after death, and an account of the morbid appearances is entered in a book kept for the purpose. In this manner, in the progress of years, a mass of facts accumulates, relating to the statistics, the types, the symptoms, the causes, the diagnosis, the pathology, and the treatment of the disease, whether successful or unsuccessful, which, both on account of the fullness and accuracy of the record, and of the extent of period it embraces, cannot but be of great value.

When these gentlemen were appointed to the office of Physician to this Hospital, they were informed that among the objects contemplated in its establishment, two things were considered of paramount importance: first, the accumulation of facts by which the true nature of fever might be more certainly ascertained; and secondly, the cautious trial of remedies by which a more sure and successful mode of treating this fatal disease might be discovered.

With opportunities of this kind for making such investigations, we might suppose that their works would abound with valuable observations and details; nor will the reader be disappointed. The works of both authors are eminently practical, and besides the history of numerous cases, and the *post mortem* appearances presented by them, we are furnished with valuable statistical accounts, calculated to shew the influence of age, sex, occupation, locality, and of the weather, upon the type and mortality of the disease, as well as in producing a susceptibility to it.

The two authors appear to have written without any concert, neither referring to the other, although their general views of pathology are very similar, and they have indiscriminately had recourse to the same reports of cases and to the same statistical accounts.

Dr. Smith, nothing intimidated by the bad success of his predecessors in this field of investigation, enters boldly into a general discussion of the nature of fever. That he was not acquainted with the difficulties and sources of error which beset his path, the following extract will abundantly show.

> When we consider how many circumstances connected with the origin and the propagation of fever are wholly unknown, which if known might have a most important influence in preventing its occurrence, in arresting its progress or in lessening its mortality; when we consider in what profound obscurity the very nature of the agents that produce it is still involved; when we consider how easy it is to swell the long catalogue of its symptoms, but how difficult it is to discriminate which, even among the most prominent of the train, are the essential and which the adventitious, and how still more difficult it is to ascertain which are the invariable antecedents and which the invariable sequents, or which the causes and which the effects; when we consider how few comparatively of the external appearances have been ascertained to be the sure and certain signs of any known condition of the internal organs, and how often the existence of several known conditions of the organs remains altogether unsuspected until the demonstration of it is afforded by inspection after death, and when finally on all these accounts we consider how vague the objects must be that are aimed at in the treatment, and consequently how uncertain, how indiscriminate, how fruitlessly inert, how perniciously active, how unsuccessful, how fatal that treatment often is, it must be admitted that fever still presents to us a vast field, in the culture of which the difficulties to be overcome are not slight, and the most diligent labor that can be bestowed upon it may by no means be attended with a sure reward.—p. 14–15.

After taking a general view of the theories of preceding writers, and painting out the erroneous views upon which their speculations are founded, he commences the investigation of the subject, by asserting, that the subject of fever cannot be understood until clear and satisfactory answers are obtained to the following inquiries:

1. What is the series of phenomena which constitutes fever? 2.

What are the particular phenomena which are common to all its varieties and combinations? 3. What is the order in which these phenomena occur in the series? 4. What are the organs, and what their states, upon which these phenomena depend? 5. What are the external signs of these internal states, or what are the indications by which their existence may be known? 6. What is the external noxious agent or agents, or the exciting cause or causes of the disease? 7. What is the particular remedy, or the particular combination of remedies which is best adapted to each state of each organ? When these questions can be clearly and perfectly answered, and not till then, we shall know the disease and its treatment.—p. 45.

What is fever? What is that combination of morbid events which enables all medical men at once to decide upon its existence, although, all have failed in assigning it a local habitation? Dr. Smith thinks that one cause of this failure is, that the attention has been confined too much to the observation and enumeration of symptoms, without inquiring sufficiently what was the condition of the internal organs which these symptoms indicated. Thus Booerhave having carefully collected all the symptoms which ever accompany fever, proceeded to strike out all such as, by their occasional absence, are inferred not to be necessary to its existence, until he reduced the whole list of essential symptoms to three; viz. chilliness, increased heat, and frequency of the pulse. Even these symptoms are denied, by later writers, to be necessary concomitants of fever; and even if they were, they afford of themselves no indications of treatment; and consequently it is necessary, if we would arrive at any satisfactory view of the subject, to push our investigations further.

Under these circumstances the following inquiries naturally present themselves: Are there any states of any organs that always exist in fever? Are these states constant? Are the organs affected constant, and can both be ascertained? The author's reply to these questions will give his theory upon the subject of fever.

The evidence is as complete as observation during life and inspection after death can make it, that a morbid change does take place in a certain number of organs in every case of fever, from the most trivial intermittent to the most alarming continued fever, from the mildest plague to the most malignant typhus: that at the two extremes of this scale, and at all the intermediate gradations of it, there are certain organs which are always affected, and that the affection in all is similar.

The identity of the organs is inferred from the indications they give of disordered function during life: the identity of the affection is inferred from the similarity of morbid appearances which they exhibit on examination after death.

The organs affected are those which constitute the nervous system; those which constitute the circulating system, and those which constitute the systems of secretion and excretion. The spinal cord and the brain; the heart and the arteries, especially their capillary extremities; the secreting and the excreting organs, which in fact are composed, essentially, of the capillary extremities of the arteries; the secreting and the excreting extremities of these arteries, especially as they terminate in the external skin, and in the mucous membranes, which form the internal skin, this is the chain of diseased organs: derangement in the nervous and sensorial functions: derangement in the circulating function: derangement in the secretory and excretory functions, this is the circle of morbid actions.

There never was a case of fever in which all these organs and affections were not more or less in a morbid state: there never was a concurrence of this morbid state, in this complete circle of organs, without fever. The events which *invariably* concur in fever, then, are a certain deviation from the healthy state in the nervous and sensorial functions; a certain deviation from the healthy state in the circulating function; a certain deviation from the healthy state in the functions of secretion and excretion. A deviation from the healthy state in one circle of actions will not present the phenomena of fever; a deviation from the healthy state in two circles of action will not present the phenomena of fever: there must be a deviation in the three circles before fever can exist. Such then are the common phenomena of fever.

But it is not the invariable concurrence of a particular number of events that is alone sufficient to constitute fever: to this must be added invariableness of concurrence in a particular order. As will be shown in the proper place, there is complete and irresistible evidence that these events do occur in one invariable order. Derangement in the functions of secretion and excretion never comes first in the series: derangement in the nervous and sensorial functions never comes last in the series: derangement in the function of the circulation never comes either the first or the last in the series, but is always the second in succession.

The order of events then is first, derangement in the nervous and sensorial functions; this is the invariable antecedent: secondly, derangement in the circulating function; this is the invariable sequent: and thirdly, derangement in the secreting and excreting functions; this is the last result in the succession of morbid changes.

Supposing the matter of fact to be as is here stated, and the proof that it is so will be adduced hereafter, it is clear that we are in possession of the true characters of fever. We know the events: we know the order in which they occur: we know therefore what it is that constitutes the disease, and we know consequently what it is by which it is distinguished from every other malady. No other disease exhibits the same train of phenomena in the same order of succession. In inflammati‹ n some of the phenomena are the same: but the order in which they concur is not the same; and this affords a clear and universally applicable mark of distinction between fever and inflammation. In inflammation there is similar derangement in the secreting and excreting functions: there is also sometimes similar derangement in the circulating function: but the derangement in the nervous and sensorial functions is seldom if ever similar: the derangement that does take place in these latter functions, while it is apparently different in kind, is certainly and invariably different in the order of its occurrence.—p. 60–63.

Admitting this to be the real condition of things, we may easily explain, from the different degrees of morbid affection in one or other of these organs, every possible variety of febrile action. Thus, at one time, the brain and spinal cord may be intensely affected: consequently the patient may be seized with violent pain in the limbs, with ferocious headache and delirium; or, on the contrary, the muscles of voluntary motion may be paralysed as in apoplexy. We may have a slighter degree of this affection, accompanied with severe pain in the head, back, and limbs, with a hot skin, a quick and strong pulse; or, instead of these, giddiness and stupor, a moderately warm or cool skin, with a frequent and feeble pulse. These two varieties are witnessed in the plague and in the common continued fever of Great Britain. We may again have that form of affection of the brain and spinal cord which is accompanied with great prostration of strength and derangement in the nervous and sensorial functions, with that general assemblage of symptoms which characterize typhus.

Under other circumstances, the disease may fall with peculiar violence upon the organs of secretion, especially upon those which belong to the digestive apparatus, and we have the different forms of fever, complicated with derangements of the liver, stomach, and intestines, which prevail in the malarious districts of hot climates.

In all these varieties of fever, these different sets of organs are invariably affected, although occasionally, the disease may invade one set so violently that we may be disposed to overlook the affection of the rest, yet a very little observation will convince us that the whole circle of organs enumerated by the author are involved in the disease.

It will easily be seen that in the comparative degree in which these organs are affected; in the shade and variety of the affection itself; in the previous state of the organs, influenced as they are by habits of life and a thousand other contingencies; in the reaction of the organs upon one another; and in the influence of the treatment upon the whole assemblage of symptoms, we have abundant means of accounting for all the endless forms of fever.

Thus, although all these organs are invariably affected in every case of fever, yet in no two cases are all these organs affected in the same degree. Sometimes one system is more affected than another; sometimes one organ of one system, and these different degrees of affection, in these different systems, are variously combined and modified. How great then must necessarily be the diversity of symptoms presented by the different forms of fever! How incalculable are the varieties that result from difference of intensity alone. One degree of affection of the brain, for example, will occasion violent head-ache, constant watchfulness, great restlessness, a peculiar expression of the eye, and intolerance of light; in another there will be no head-ache, or none of which the patient will complain; there will be sleep though it be disturbed and unrefreshing; there will be no peculiar expression of the eye, and no intolerance of light. By one degree of affection the sensibility will be rendered preternaturally intense; by another it will be totally obliterated: one will produce violent delirium, another, only slight wandering, or unrefreshing slumber: one, violence requiring restraint; another, profound coma. In the circulating system the symptoms will alike vary. One degree will produce a quick, strong and hard pulse; another, a quick, small and feeble pulse, another, a slow and intermittent pulse. A similar diversity will be found in the temperature of the body: in one, the heat will be little changed; in another, it will be below the natural standard; in a third, it will be intense, and the organs of secretion and excretion will equally vary in the extent of their morbid changes.—p. 69–70.

The reader will now be prepared to appreciate the following remarks of the author upon the importance, in endeavoring to form a just view of fever, of looking beyond the symptoms to the internal states of disease of which they are the sign:

That no two cases of fever can ever be precisely the same, and that it must be vain to seek for the common phenomena of the disease in the external symptoms, must now be obvious: and why success can never attend the search after these common phenomena in such symptoms as "shivering, frequent pulse, heat," must be equally manifest. These as well as all other symptoms depend upon the state of the organs. But we have seen that in one degree of the same affection of the same series of organs there may be shivering; excited pulse; burning heat; while in another degree there may be no shivering, a slow pulse and a cold skin: so that from one and the same affection, differing only in the degree of its intensity, the symptoms may not only vary but be directly opposite. The proper object of pursuit in all these inquiries, therefore, is the real nature of the affection, and the symptoms are of consequence only as they are indications of the existence of that affection. Symptoms are not *the* thing in which observation should terminate, but signs of the thing without the knowledge of which, in every individual case that may come under his care, the practitioner ought never to be at rest, and to the discovery of which they serve as guides.—p. 70–71.

Dr. S. objects to the general division of fevers into idiopathic and symptomatic. In his view, as we have already seen, the fever of inflammation is a very different thing from true fever—accompanied with a different train and order of symptoms, and, therefore, requiring a different classification and name.

The division of idiopathic fevers again into intermittent and continued, he objects to, as founded upon a fact in the highest degree trivial. The general division of continued fever into synocha, synochus, and typhus, he asserts is founded upon no principle which could lead to the formation of the assemblages of symptoms which are given as characteristic of these diseases, or to their nomenclature when thus assembled; while, at the same time, the symptoms thus enumerated do not constitute the disease, nor convey any adequate conception of its nature.

A further objection which appears to us in every respect valid, is drawn from the exclusion of the exanthemata from the order of fevers. These diseases, as the author very justly asserts, however they may be modified by the cutaneous affection, are essentially fevers, exhibiting the same series and order of morbid derangements, and the same assemblage of symptoms. It is doubtless proper that these varieties should be discriminated and named, but this should be done with a distinct understanding that they constitute merely varieties of one great form of disease, distinguished by circumstances which modify its features without changing its identity.

This mode of viewing fever as one great and extensive malady never differing in nature, but in every two cases differing in intensity, and giving rise by these differences in intensity to various forms of disease, thus affords a principle of arrangement applicable to all those various forms, which, while it is at once simple and comprehensive, is at the same time in the highest degree practical. It directly leads the mind to the observation of the real, the important differences that exist or that may arise; those differences which must influence and guide the treatment, if it be not altogether blind, and in the worst sense of the term empirical. This principle might easily be extended, and I think with advantage, so as to comprehend the Exanthemata, and all the forms of fever which have hitherto been known to exist, or which can arise. Scarlet fever, for example, is continued fever attended with a peculiar eruption upon the skin: at one time it occurs in a mild, at another in an exceedingly severe form: the assemblage of symptoms in the first are precisely those which it is intended to comprehend under the term synochus: the assemblage of symptoms in the second are those which are designated by the term typhus: thus scarlet fever exhibits at one time the synochoid, and at another the typhoid type; the first being what is commonly termed scarlatina benigna, the second scarlatina maligna; and each type is capable of existing in two degrees of severity, one of which may be conveniently distinguished by the term mitior, and the other by that of gravior.

In like manner small-pox is a fever attended with a peculiar eruption upon the skin, which eruption modifies the disease in a very remarkable manner and gives it a history and progress peculiarly its own, but it is as much a genuine fever as typhus, and ought no more to be taken out of this class on account of the eruption upon the skin, than scarlatina, which likewise modifies, in a very considerable degree, the whole train of febrile symptoms, and is attended with a peculiar condition of some exceedingly important internal organs. Small-pox, like all the diseases of this class, occurs in two widely different forms; the one mild, the other intensely severe: in the first the concourse of symptoms are precisely those of the synochoid, in the second of the typhoid type. And the same I am satisfied is true of the plague, of the yellow fever, and of all the different forms which this great disease, of many aspects and names, but of one uniform and unchanging nature, presents.—p. 85–87.

We cannot agree with our author as to the perfect futility of the distinction of fevers into intermittent and continued. It is true that we are unable to trace the connexion between the cold and hot stage of fevers; it may be equally true that the condition of the internal organs upon which the difference depends, is unknown to us, but when we see that these conditions are as uniform as the cause of the fever and the climate in which it occurs, and that they are not accidental symptoms, but founded upon different states of the system, and requiring a radical difference of treatment, we cannot say that the distinction is without foundation. Nor can it be said that the type appears to be determined by the intensity of the disease, since the most violent forms of yellow fever still preserve their intermittent character, while the milder forms of intermittent fever often approach to the continued type without exhibiting any peculiar malignity.

After this general outline, the author enters into a general history of the different forms of continued fever, in which he attempts to prove, by an analysis of the symptoms, the truth of the positions he has laid down.

In the milder forms of continued fever (synochus mitior, in the language of Dr. S.) the first symptom of the appproaching disease is "loss of mental energy, indistinctness, and consequent confusion in the trains of ideas, and inability to attend to their relations." Connected with this is "loss of energy in the muscles of voluntary motion," evinced by lassitude and muscular debility. The next symptom in the order of succession is still moɹe characteristic, and is denominated by the author "febrile uneasiness"—that peculiar feeling of restlessness and anxiety which so frequently precedes a febrile attack. These symptoms are followed by pain in the back or loins, and then in the limbs. The surface of the body now begins to manifest its participation in this general debility "by its sensitiveness to the agents by which it is surrounded, and by its inability to resist their influence," and chilliness and shivering, symptoms not depending upon cold, but upon diseased sensation; since it exists in a warm room, and when the heat of the body is above the healthy standard.

These symptoms Dr. S. thinks are clearly referrible to derangement of the function of the spinal cord and brain. Very soon the vascular system and the respiration begin to be affected, though as yet, it is an affection which proves them to participate in the debility which pervades the rest of the system—the pulse being quick and weak, and the respisation short and hurried.

After an interval, longer or shorter according to circumstances, the vascular system partakes in the disease in a different manner, the pulse becomes more frequent and stronger, and the skin, which was before cold, becomes preternaturally hot. As soon as this change takes place in the circulation, the functions of secretion and excretion become deranged; the tongue is covered with fur; thirst comes on; the secretions of the liver and of the whole alimentary canal are vitiated; the urine is altered in appearance; and the skin is dry and parched.

At this period, then, the fever is fully formed; the series of morbid phenomena is complete: any thing more that happens is referrible to degree and to duration, and must be the result of one or other of these circumstances, or of their combined operation. And we now see that the organs affected, constitute precisely that system of organs which has been described as forming the febrile circle: that the symptoms which denote the fever are just the symptoms which indicate a derangement in the several functions performed by these organs; and that the order in which they become successively involved is exactly that which has been assigned.—p. 97.

The fever being now fully formed, there is as yet no local inflammation. It does, however, frequently, perhaps generally, supervene, and to its presence the author attributes the difference between the milder and the more severe grades of fever. The organs thus affected are the spinal cord, the brain, and their membranes, the mucous membrane of the lungs, and the mucous membrane of the intestines. From the modifications produced by the affection of these organs and tissues, all the different varieties and innumerable symptoms of fever are produced; and, if so, it becomes a matter of great importance to ascertain the symptoms by which these internal derangements are indicated. From the seat of the local affections, Dr. S. divides continued fever in the following manner: *Synochus with cerebral affection—Synochus with abdominal affection—Synochus with thoracic affection*,—and lastly, there is a *mixed form* of the disease in which all these organs are simultaneously affected. Of the prominent symptoms of each of these we shall give a short abstract.

1. The most important symptom of cerebral affection is pain. Pain, however, although generally present, is not uniformly severe, and it is not uncommon for the most unequivocal and extensive changes of structure to take place without the pain having been so violent as to attract much attention.

2. In some cases the pain is absent entirely. Giddiness is then the substitute.

Now and then no pain whatever is felt. Question the patient as much as you please, and he will tell you that he never has felt any pain. In this case giddiness is the substitute. Giddiness in the commencement, and in the early state of fever, is as certain a sign of cerebral affection as pain. Striking illustrations of this are afforded by several cases detailed in the pathology; by consulting which, the reader will see that precisely the same morbid changes take place in the structure of the brain, although nothing but giddiness be complained of, as occur in those which are attended with the acutest pain. The practitioner will therefore fall into a fatal error who is seduced into security because pain is absent; and who neglects the remedies proper for inflammation of the brain, because the patient complains only of giddiness. If giddiness be combined with pain, or alternate with it, which is not uncommon, the giddiness being slight if the pain be severe, and the pain being slight if the giddiness be distressing, it indicates a more severe affection than if either exist alone.—p. 110.

3. A dull and heavy expression of the eye. The conjunctiva sometimes becomes brighter and more glistening than natural, or its vessels become injected.

4. There is, at the same time, an increase of general sensibility, particular in the senses of sight and hearing.

5. The countenance is indicative of suffering; though, without an expression of anxiety at the commencement of the disease.

6. So long as the pain in the head, giddiness, and increased sensibility continue, there is *sleeplessness* and restlessness.

7. To these symptoms delirium is added, sometimes as the forerunner of a favorable change, more frequently, as an indication of the increasing violence of the disease.

8. The pulse, thus far, may not materially be altered; neither does the state of the tongue or of the evacuations materially differ from what is witnessed in the milder forms of the disease.

If the disease proceeds unfavorably, after some days, an entirely new train of symptoms sets in.

1. The pain in the head or the giddiness diminishes, often disappears altogether; or if any uneasiness remain, it is a sense of dulness and heaviness. The period at which this change takes place is about the 10th day, or earlier, if the attack be violent.

2. Simultaneously with this there is diminution of the general sensibility; the mind becomes dull; and the eyes injected.

3. Delirium at this time supervenes, or if this be absent, there is moaning and incoherent muttering during the short and disturbed slumbers of the patient.

4. The pulse now becomes increased in frequency, and at the same time, becomes weaker.

5. And lastly, signs of disease in the chest and abdomen begin to appear; a case purely cerebral, from the commencement to the termination, being rarely met with.

These symptoms are followed, towards the termination, with those undisguised marks of cerebral congestion with which every medical reader is familiar. If the disease of the brain be more acute, the general train of the symptoms is very similar, except that they succeed each other more rapidly; and, if the disease be not arrested, the fatal termination takes place more speedily. Among the unfavorable symptoms in this form of disease the author mentions intense pain in the head or back with a *slow* or *intermitting pulse*, and frequent sighing. Among the favorable symptoms, an easy quiet slumber, which very generally is the harbinger of immediate convalescence.

The second form of fever is that combined with *Thoracic Affection*. Upon this affection the author remarks, that although a very severe form of fever may be sometimes accompanied with a slight degree of thoracic disease, yet "whenever the thoracic symptoms are sufficiently intense to become prominent, and especially when they occur early, or attend on the commencement of fever, they invariably and very considerably aggravate the general febrile symptoms." The symptoms of this affection we shall leave the author to describe in his own language:

Pain in the chest, sometimes severe, sometimes only slight; sense of stricture or dyspnœa; inability to expand the chest by a full inspiration without pain or uneasiness; cough frequently aggravating the pain; sometimes dry, sometimes accompanied with frothy mucous expectoration. Respiration sometimes slow and heavy, at other times, on the contrary, short and quick; never natural: perhaps the physician may detect thoracic disease in the more obscure, and measure its extent in the more obvious cases, by observing the manner in which the patient breathes, better than by any other single means. The altered respiration is very frequently accompanied with that peculiar noise in breathing which is termed "mucous rattle."—p. 133.

This condition of the lungs very generally brings on stupor or low delirium at an earlier period than it would otherwise appear. Violent delirium, he asserts, is seldom, if ever, found in combination with thoracic disease.

The third variety, or fever combined with *Abdominal Affection.* Abdominal disease in fever exists under two forms. It may come on at the commencement, and give early indications of its presence, or it may come on later, and then, although the affection is equally severe, it will be more difficult of detection.

1. The first symptom is nausea, with retching or vomiting. These symptoms are generally enumerated among the ordinary attendants on fever; but, as they are rarely present in a case decidedly cerebral or thoracic, the author considers them as evidence of an abdominal affection more severe than usual.

2. At the commencement, the bowels are generally constipated, but in a day or two looseness comes on. The concurrence of this symptom with the preceding is an infallible indication of the presence of an abdominal affection, that is likely to render the case formidable.

3. Pain, of which the patient complains spontaneously. This however is by no means an invariable attendant. Sensibility of the epigastrium to pressure is a much more frequent attendant. The pain which arises spontaneously ceases after a few days, although there may still exist extreme sensibility upon pressure, upon which fact the author makes the following remarks, which are not unimportant in considering the pathology of fevers.

There is thus a remarkable coincidence between the progress of the symptoms in the abdomen and in the head. We have seen that however intense the cerebral affection, the pain of the head which accompanies it diminishes after a certain time, and in a day or two after it has begun to diminish, ceases altogether. In like manner the pain which ushers in an acute abdominal affection diminishes after a certain time, and soon wholly disappears. After this period, therefore, we should have no more indications of abdominal than we have of cerebral pain were the intestines, like the brain, enclosed in a bony case. When an organ can be touched, it gives us an additional and an invaluable means of ascertaining its morbid condition: and this is one reason why that condition is commonly so much more certainly known in surgical than in medical diseases. What the result would be, could we press the brain as we can the abdomen, after its sensibility is so much diminished as to cease to occasion pain, we do not know; but it would be a bad use indeed to make of the additional means afforded us of ascertaining the condition of the intestines, were we to allow the additional information we thus gain, to obscure our perception of the perfect analogy there is in the progress of both affections. We know that as the disease advances in both, the pain ceases; but, in the one case, we have the means of ascertaining that there still remains preternatural tenderness on pressure, as in ordinary inflammation, which we are without the means of discovering in the other: still the important practical fact afforded by the history of both is the same, that disease having reached a certain point, the pain diminishes; and having advanced still further entirely disappears.—p. 143–4.

4. The appearances of the tongue.

Together with these decisive signs, which alone are abundantly sufficient to enable us to ascertain the presence of the affection, we have an additional and an exceedingly valuable guide in the peculiar state of the tongue. In these abdominal cases, the tongue is preternaturally red. Sometimes this increased redness is of a bright and vivid color, and pervades the whole tongue; at others, it is confined to the edges or to the tip, and it is usually remarkably apparent in the latter. While thus vividly red, the body is often loaded with fur; the color of the fur is often of a dirty white or greyish color; but, perhaps, while the edges and the tip are thus intensely red, the most usual color observed on its body is that of a dirty yellow. In these cases, the papillæ appear much enlarged, and are seen prominent through the fur, vividly red. In this condition of the tongue it always remains moist for some time, and it is not attended with urgent thirst; but, as the intestinal disease advances, the tongue gradually becomes less vividly red and more dry, and as these changes go on, the lips and teeth often become sordid.

Instead of being from the commencement of a vivid redness, the color of the tongue, in other cases, is of a darker and duller tint; there is less fur upon the body, and that which covers it is of a dirtier and darker tinge; this state of the tongue is always attended with greater thirst: it is apt to become more and sooner dry, and, at the same time, the lips and teeth become more and sooner sordid.—p. 145.

5. The pulse in this affection exhibits no very striking or distinctive character—its common range being from 80 to 100, beyond which it seldom rises until near the termination of the disease.

6. After a time the purging ceases; though without a favorable change in the general symptoms, this is not to be considered as an indication of approaching convalescence.

The fourth variety of fever is founded upon the complication of disease in all the organs above enumerated. Whenever, Dr. S. remarks, a severe case of fever occurs without any striking prominence of affection in any organ, and when, on examination, there are found indications of severe disease in all, a very formidable case is certainly to be expected. Upon this subject we extract the following remarks, which will go far to counteract the very prevalent error that severe disease is not likely to take place in two different organs at the same time.

Whatever be the number of organs simultaneously affected, the nature of the affection in each is always the same, and is not in the slightest degree changed by the complication. Disease in the brain is the same, whether the brain alone be prominently affected, or the brain and intestines, or the brain, the intestines and the lungs. Each organ is liable to its own specific disease, and that disease goes on with the utmost regularity, whether it be the sole organ so far diseased as to suffer a change in its structure, or whether many be simultaneously affected in the same manner.

In like manner the symptoms, when any symptoms are present, are essentially the same, whether the disease exist alone, or whether it be complicated with several others. The symptoms of inflammation of the brain are the same, whether cerebral inflammation alone be present, or whether it be complicated with inflammation and ulceration of the mucous membrane of the intestines. And the symptoms of inflammation and ulceration of the mucous membrane of the intestines are the same, when any symptoms are present, whether these affections exist alone, or whether they are complicated with cerebral inflammation. The occasional absence of symptoms in the subordinate organs, overwhelmed by the preponderence of affection in the principal is a proof that they are subordinate. It would, therefore, be useless to detail the symptoms which occur in the mixed cases, since they must only be a repetition of those which have been already enumerated. Their concurrence in individual complications, and the modifications which they undergo from such particular combinations, will be best understood from the study of the cases.—p. 156–7.

Chapter IV. is devoted to the consideration of typhus fever. Dr. S. remarks that although the symptoms of typhus and synochus are so strikingly different, yet the affection of the organs is the same both in their nature and succession. We shall merely notice some of the prominent features by which typhus is characterized.

There can be no question that, from the very first commencement of the attack, as well as through the whole course of the disease, the prostration of strength, both physical and mental, is greater in typhus than it is in synochus.

The chilliness is, upon the whole, greater and longer-continued than in synochus: yet there is less constantly shivering, and the heat, when it succeeds this state of chilliness, is seldom as great as in the latter; while there are cases in which the heat never exceeds the natural standard.

The febrile uneasiness is greater; the restlessness is incessant; the face is pallid; the features are shrunk; the expression of the countenance is most peculiar; it is strikingly indicative of weakness and suffering; the experienced eye can tell at a single glance, even at this early period, to which of the two types that countenance belongs. The pulse is always weaker and more rapid than in the corresponding stage of synochus.

There are cases in which the pain of the head is equally severe as in synochus: but this may be justly considered as rare. In general it is less acute. Dulness, confusion, stupor, giddiness, are more common than severe pain, and are often the substitutes for it. Though some degree of pain be generally present, yet it is by no means uncommon for one or more of these sensations to occupy its place completely.

When pain is present it diminishes sooner and disappears more completely than in synochus: when it is not present, the advancement of the disease is indicated by increasing insensibility, and by the rapid transition of dulness or confusion into a state of stupor approaching to coma. The eye is already muddy, and it soon becomes injected and suffused.

There is little or no sleep; the restlessness is great; there may be no violence; but there is abundance of inquietude.

Delirium is more constantly present than in synochus; and when it comes it comes earlier: its presence is not unusual as early as the sixth or seventh day; and it may appear still sooner, but that is rare. It consists of low muttering incoherence rather than of loud and violent talkativeness; and is expressed in moaning rather than screaming.

Like delirium, muscular tremor is much more constantly present in typhus than in synochus; and its relation to delirium is so close that it is sometimes observed to supervene on the very same day; frequently on the day following; and, if it appear at all, it is seldom longer absent than the third.

The respiration is often not very obviously affected, but if it be attentively observed it will usually be found to be shorter and quicker than natural.

The tongue is always foul on the first or second day; it seldom continues moist longer than three or four days; it is often quite dry as early as the fourth, especially on the body and at the root.

During its progress, erysipelas, first appearing on the face, then extending over the scalp, and often down the shoulders and back, is very apt to occur. Excoriation on the back and hips often form sloughing sores of great malignity and extent, while enlargement, inflammation and suppuration of glands situated in different parts of the body frequently appear.

Typhus terminates much earlier, whether favorably or unfavorably, than synochus.—p. 162–7.

### Dr. S. vehemently objects to the opinion which attributes the prostration of strength in the violent forms of typhus, to debility.

It is remarkable, and it is highly characteristic of these intense forms of disease, that their pathology exhibits a striking contrast to that of the less severe affections. No morbid appearances are visible in the organs which seem capable of accounting for death. There are signs of vascularity; the vessels are turgid with blood, and consequently the organs on which they are spent are in a state of congestion. But they seldom if ever exhibit any real appearance of inflammation, and still less do they contain any true inflammatory product. Why? Not on account of debility; but because the force of the disease is so great as to overwhelm the powers of life at the first onset, allowing even of no reaction, and much less of that continued excitement which is part and parcel of the inflammatory state, and which is indispensable to an inflammatory product. Reduce the intensity of the disease a little, bring it just within the limit that is compatible with the continuance of life for a given time, and then the products of inflammation at once appear in the greatest possible purity, variety, and extent.—p. 177-8.

An opinion true, perhaps in general, but to be received with very great qualifications. Even upon his own principles, the affection of the nervous system may be so violent as to paralyze the organs at once, and interrupt their functions. If, therefore, to this influence be added exposure, fatigue, bad living, anxiety, and confined air, why may not a form of fever prevail whose characteristic feature shall be debility rather than inflammation?

We shall close this part of the subject with a brief account of some of the most prominent appearances after death.

*Morbid appearances in the Head.*—Of the membranes of the brain, the arachnoid is the most constantly diseased. It is either more vascular than natural, or is altered in structure, being thickened and opake, and when in this condition generally has a gelatinous fluid effused beneath it. It is also often adherent to the membranes, both above and below. The dura mater is affected in a similar manner, although less constantly. The pia mater is seldom changed in structure, though it is generally preternaturally vascular. Of the changes which take place in the substance of the brain we quote the following:

The brain itself is seldom or never in a healthy condition; the morbid changes to be distinguished in it differ greatly in degree in different cases, but still, in almost every case, some morbid change is to be discerned. These changes consist of an altered state of its substance, or of its cavities, or of both. The most usual change apparent in its substance is a higher degree of vascularity than natural. This increased vascularity is sometimes confined to the surface; sometimes it is more manifest deep in its substance; and, while common to both, it may exhibit different degrees of intensity in either. When on the surface, this preternatural vascularity is denoted by a greater fulness of the vessels, and, apparently by an increase in the number of bloody points, which are rendered visible by an incision with the scalpel. And in both situations it may exist in all degrees, from a faint blush to a deep and vivid redness. The substance itself is sometimes softer, sometimes firmer than natural. The softening differs in degree and in extent. Sometimes the entire cerebrum is manifestly and considerably softer than natural; at other times, only particular portions of it are found in this softened state. Now and then, but very rarely, abscess is discovered within its substance. It is remarkable that the cerebellum is always considerably softer than the cerebrum: whence these two portions of the organ are often observed to be in opposite states, the cerebrum being frequently preternaturally firm, and the cerebellum being almost always softer than natural. The pituitary gland also is very constantly softened, and often in a state of suppuration. When the cerebellum is preternaturally firm, the firmness is usually general.

It is observable that the two morbid conditions now described, that of excessive vascularity and that of increased secretion, are never co-existent. If the vessels of the brain and its membranes are loaded with blood, there is little or no fluid within the former or between the latter: if, on the contrary, the effusion be great, there is little or no appearance of vascularity. Effusion is the effect and the termination of vascularity: it is the ultimate result of vascular action, and the effect having ensued, the cause ceases to be apparent.

The substance of the spinal cord is seldom changed, either in vascularity or in consistence: the morbid changes which this organ undergoes have hitherto been observed only in the membrane that invests it, which, as has been just stated, is not only highly vascular, but likewise contains a much larger quantity of fluid than natural.—p. 195–6.

*Morbid appearances in the Thorax.*—The principal morbid appearances to be observed here are in the lining membrane of bronchi, consisting of a preternatural redness, specific and uniform, as much darker than what is observed in ordinary inflammation as is the difference between venous and arterial blood.

This darkness of color apparent in the bronchial lining, increases in degree as the tubes of the bronchi diminish in size: while it may be only just discernible in the large trunks, the color may be nearly black in the minute branches. This change in the natural color of the membrane is indicative, not only of an increase in its vascularity, but of alteration in its structure. It is almost always attended with a preternatural thickening of its substance, as is demonstrated by cutting through the tube and reflecting the membrane. The tubes themselves contain more or less fluid, which consists of mucus, mixed with pus. Analagous to what has been stated with regard to the vascularity of the brain and to its secretions, when the quantity of secretion contained in the bronchial tubes is great, the degree of vascularity apparent in the membrane is lessened.—p. 197.

The description of the morbid appearances exhibited in the air passages after death from scarlatina, a disease that has of late been very prevalent and fatal in many parts of this country will not be uninteresting to our readers.

In scarlet fever, the morbid changes are somewhat different. The mucous membrane covering the trachea, the larynx with its cartilages, the amygdalæ and the soft palate is inflamed; the redness is of a brighter and more vivid color than that which has been stated to be characteristic of continued fever without an eruption: it is similar to the characteristic color of the scarlatina tongue. But what is very remarkable, and what appears to justify the view we have taken of scarlatina and the division we have suggested of its types, when the cases are severe, the color of the mucous membrane becomes much darker, the deepness of the tinge increasing with the severity of the affection, until, at length, the color closely resembles that which is peculiar to ordinary fever.

As in continued fever without an eruption, so in scarlatina, the increased vascularity of the mucous membrane is accompanied with a preternatural thickening of its substance. In scarlet fever, that portion of it which covers the epiglottis, the rima glottidis, and the arytænoid cartilages, is especially found in this diseased condition. When this inflammation and thickening passes into the state of ulceration, which it often does, the arytænoid cartilages are the special seat of this process, although the ulceration often extends to the amygdalæ, and sometimes to the root of the tongue.

When in every other respect healthy, the substance of the lungs in fever is so constantly found either engorged with blood or infiltrated with serum, that these changes would seem to form essential parts of the morbid phenomena.—p. 197–8.

*Morbid appearances in the Abdomen.*—The great importance which has been attributed to inflammation of the mucous membrane of the stomach and intestines, as the cause of fever, will justify an extended extract upon the subject.

On opening the cavity of the abdomen all the viscera contained in it appear, in general, more vascular than natural, and invariably of a darker color than in the state of health. Several of the organs are affected in a uniform and peculiar manner, but that which is by far the most constantly diseased is the mucous membrane of the small intestines; and especially that portion of it which lines the ilium and the cæcum.

The varieties of disease exhibited by this membrane may be comprehended under three, namely, vascularity, thickening, and ulceration.

In all cases increased vascularity is the first stage of disease: in a great proportion of cases this increased vascularity is confined to the inferior extremity of the small intestines, which is often distinctly inflamed when not the slightest deviation from healthy structure is traceable in any other part of the canal.

The second stage of disease consists in thickening of the membrane, or in deposition of matter beneath, or in both. Preternatural thickening of the membrane is often of very considerable extent: deposition of matter beneath it appears to be confined to the situations of the mucous glands. These glands are found in all states and stages of disease from the least to the greatest enlargement, and from the mere abrasion of their surface to the entire ulceration of their substance.

The third stage is that of ulceration, which may supervene when the membrane is affected in either of the modes just described; but the ulcer will not be the same in both cases: in each it will have a different and a distinctive character. If ulceration take place while the mucous coat is in a state of simple vascularity, the ulcer will in general be extensive but superficial; its surface will present a smooth appearance, and its margin will be regular and defined: if, on the contrary, it occur after thickening of the membrane or enlargement of its glands its characters will be just the reverse: it will be less extensive, but more deep, because it must penetrate a mass of adventitious matter before it can reach the other coats; and, for the same reason, its margin will be more elevated and its surface more ragged. It is in this form of ulcer that perforation of the intestine generally occurs; in which case the mucous and muscular coats alone are ulcerated: the peritoneal gives way from gangrene.

Whenever the mucous membrane is ulcerated, whatever be the form of the ulcer, the corresponding portion of the peritoneal coat is more vascular than natural; and perforation must be attended with inevitable death, on account of the extensive and intense peritonitis excited by the escape of fæces into the peritoneal cavity.

Proportioned to the extent and degree of these changes in the intestine are, inflammation, enlargement, induration and suppuration of the mesenteric glands: and invariably those glands which are embedded in that portion of the mesentery attached to the affected intestine, are the most diseased.—p. 199–202.

It has been our intention to give a fair view of the author's opinions upon fever, with such of his illustrations as would enable our readers to derive practical benefit from the facts which the author has advanced in support of his speculations. We shall conclude the subject with the following summary which he gives of his doctrine:

In conclusion, then, the doctrine of fever which appears to approximate most nearly to the truth, may be summed up in a few words. The immediate cause of fever is a poison, which operates primarily and specifically upon the brain and the spinal cord. The diseased state into which these organs are brought by the operation of this poison, deprives them of the power of communicating to the system that supply of stimulus (nervous and sensorial influence) which is requisite to maintain the functions of the economy in the state of health. The organs, the seats of the functions, deprived of their supply of nervous influence, become deranged, the derangement in each taking place in a fixed order, and in a determinate manner. Subsequently to the nervous and the sensorial, the organs the next to suffer are those of the circulation; then those of respiration; and, ultimately, those which belong to secretion and excretion. The condition of the nervous system which produces this derangement in this circle of organs, occasions further, in that portion of the circulating system which consists of the capillary blood-vessels, that peculiar state which constitutes inflammation: hence inflammation is almost always established in one or more of the organs comprehended in the febrile circle, and sometimes in all of them. The peculiar and primary affection of the nervous system, which is here assigned as the cause of inflammation, does not become identical with inflammation, but superadds the morbid condition of inflammation to its own; does not lapse into or terminate in the inflammatory state, but accompanies it, and by this combination modifies in a peculiar manner the inflammatory process.—p. 358–9.

Such are the author's views upon the subject of fever. Are they satisfactory? Has he arrived at any thing certain and definite as to the order and succession of the diseased action? We think not. Upon what authority does he attribute the primary symptoms to the brain and spinal cord? Admitting that these organs are effected, what reason has he for asserting that their affection is not of the same nature, and proceeding from the same causes with that of the organs whose symptoms are exposed to view? The organs of the nervous system are supplied with vessels and tissues like the other organs; if the debility of the latter is to be traced to derangement of the former, to what is the derangement of the former to be attributed? The poison which produces fever acts upon the nervous system; may it not act sympathetically upon the branches, as well as upon the trunk, and thus simultaneously affect the whole?

Let us enquire a little into the true condition of things. Is it true that the affection of the secretions is the last in the train of deranged action? An individual complains of the listlessness, languor, debility, &c., which indicate the approach of fever. What is the cause of these symptoms? Dr. S. says that the supply of nervous energy is cut off by an affection of the brain—others say that the capillary circulation is deranged and the secretions are consequently interrupted. They appeal to the furred tongue, the pale alvine evacuations, the loss of appetite, &c. What is the practice? An emetic or a mercurial cathartic is given; the liver resumes its functions, the appetite returns, the skin resumes the glow of health, and the vigor of the body returns at once. Will the author pretend to say that the medicine acts by immediately restoring nervous energy?

We of course estimate his theory but lightly. This, however, does not detract from the value of the great body of practical information which he has so patiently collected and so judiciously arranged. On the contrary, if his theory has added any thing to this part of the work, we could easily forgive its defects, even were they much greater than they are.

With a few remarks upon the course of treatment pursued by these gentlemen, we shall close this article.

The simple forms of fever, Dr. S. remarks, require little or no treatment; but as the severer forms derive their danger, in his opinion, from local inflammation, bleeding is his sheet anchor, and is to be resorted to the extent of reducing the inflammation. The amount necessary to be taken, however, will be less than in pure inflammation.

Mere relief of inflammation is nothing; to render a severe inflammation a less severe inflammation is to do nothing; because the less severe inflammation may be fatal just as certainly as the more severe: the inflammation must be subdued, or the case, if not wholly lost, becomes dangerous and doubtful.

The abstraction of blood must be carried to the extent of subduing the inflammation: there is no other limit to the quantity to be taken but that which is adequate to subdue the inflammation. To attempt to measure the quantity by drachms or ounces is wholly vain; because, if the remedy be properly employed, the quantity will vary in every individual case.

The tendency of the preceding observations is not to countenance frequent and large abstractions of blood in fever, but to save the blood of the patient, by taking the due quantity at the proper time. Smaller bleedings will subdue febrile than pure inflammation. Febrile inflammation, as has been so often stated, is a modified inflammation, the modification consisting in less activity in the vascular system and greater depression in the nervous. Whence a moderate bleeding will make an impression upon febrile inflammation which can be equalled in pure inflammation only by a large bleeding. He who takes away sixteen ounces of blood in fever adopts a bolder and more decisive practice, and brings more effectual relief to his patient, than he who abstracts thirty ounces of blood in some other forms of inflammation; and he who takes away six ounces of blood in one febrile case, does more than he who takes away sixteen in another. But the question never can be whether the bleeding should be small or large: that is nothing. The thing to be considered is the condition of the organs, the state of the system; not the ounces of blood to be taken, nor the number of periods at which it is to be removed. Abstract blood to the subdual of the inflammation—that is the rule; abstract blood at the very commencement of the inflammatory action; if you are in time to do it, at the very commencement of the febrile excitement.—p. 395–7.

When this is effected the physician has nothing to do but look on and see the patient get well. To purgatives he attaches very little importance beyond the simple operation of procuring three or four daily evacuations by rhubarb or castor oil, with two or three grains of calomel every other day.

As an adjuvant to bleeding where it fails to relieve the brain, they recommend the application of cold water to the head in the form of the "cold dash."

The mode of applying this remedy has been already described in the present number of this Journal, by Dr. A. Judkins. Dr. S. asserts that no degree of inflammation can withstand the efficacy of this application; under its use the pain subsides, the countenance becomes pale, and the pulse is reduced to a mere thread. It is not intended of course to supercede the use of subsidiary measures for the reduction of inflammation, such as blood-letting, purgatives, &c. It will be obvious also that so powerful a remedy must be used with some regard to discretion.

This is represented as being infinitely superior to the application of ice; and, it is said, that three or four repetitions will commonly suffice to subdue the most intense cerebral affection.

The following remarks upon the use of tartar emetic in the treatment of fever combined with affections of the lungs will be considered novel; this medicine, in large doses, having been principally confined to the treatment of *inflammation* of the lungs.

Fortunately, there is a remedy nearly as powerful and efficacious in intense thoracic affection, as blood-letting and the cold dash are in the cerebral. In the severe bronchial affection of fever, blood-letting is of little avail. It seems to have scarcely any control over the peculiar affection of the lining membrane of the bronchial tubes, or even over the inflammation of the substance of the lung, which so often accompanies the intense form of thoracic disease. It weakens the patient without making a decided impression upon the disease. Laennec states that the pathology of pneumonia could scarcely be learnt under his practice; for that he treated the disease, not by blood-letting, but by tartar emetic; and that all his patients recovered. I thought this one of the exaggerated statements in which medical writers sometimes delight to indulge; but it immediately occurred to me that this remedy might prove exceedingly efficacious in the bronchitis of fever. Its efficacy has surpassed my expectation. It seldom fails if exhibited with promptitude and decision. The mode in which it is most efficiently administered, is in doses of two grains, dissolved in an ounce of water, and repeated every second, third, fourth, or sixth hour, according to the severity of the case.—p. 415–6.

The last chapter of Dr. Smith's work is devoted to the subject of *relapses* in fever. A large proportion of those who die in consequence of fever, perish from imprudence or inattention after the danger of the disease, under judicious management, is past. This fatal result, Dr. S. asserts, takes place generally from rising out of bed to early, or from the injudicious use of animal food. After a severe attack of fever, patients, weary of their long confinement, are seldom aware of the extent of the debility which pervades every part of their system; and, under the excitement of cheerful society, are insensible to fatigue until irreparable and fatal injury results from exertion. The practitioner cannot be too cautious in the directions he gives respecting the exercise of a convalescent patient, nor should he be permitted to shelter himself from censure when unpleasant consequences result, by attributing them to the imprudence of the patient.

The second most frequent cause of relapse is imprudence in diet, especially, in the use of animal food. The ravenous appetite which generally attends convalescence from fever renders a great deal of self-denial necessary to prevent the patient from injuring himself by excess. Unfortunately the prejudice of the public respecting the necessity of an abundant supply of nutritious food to recruit the wasted energies of the system so far coincide with the calls of appetite, as to render the prudent advice of the physician too often unavailing.

Dr. S. enumerates three cases in which the danger of relapse is peculiarly imminent. 1. When the disease has run its course with great violence. 2. When the disease has been overcome by copious blood-letting. Convalescence has thus been brought about which will be permanent, provided, proper prudence and precaution are observed, but such a state of irritability has been induced, that the most unhappy consequences result from the least imprudence. 3. When from the presence of irritation, or unsubdued inflammation, the convalescence is tedious and protracted. Under these circumstances, therefore, the practitioner should be doubly on his guard, and not consider that his duty has been discharged, without prescribing a strict system of diet and regimen, and faithfully warning the patient and his friends of the consequences of departing from it. F.

